# Deficits in N-Methyl-D-Aspartate Receptor Function and Synaptic Plasticity in Hippocampal CA1 in APP/PS1 Mouse Model of Alzheimer’s Disease

**DOI:** 10.3389/fnagi.2021.772980

**Published:** 2021-11-30

**Authors:** Le Xu, Yiying Zhou, Linbo Hu, Hongde Jiang, Yibei Dong, Haowei Shen, Zhongze Lou, Siyu Yang, Yunxin Ji, Liemin Ruan, Xiaoqin Zhang

**Affiliations:** ^1^Zhejiang Key Laboratory of Pathophysiology, Department of Pharmacology, School of Medicine, Ningbo University, Ningbo, China; ^2^Department of Psychosomatic Medicine, Ningbo First Hospital, Ningbo Hospital of Zhejiang University, Ningbo, China; ^3^Key Laboratory of Addiction Research of Zhejiang Province, Ningbo Kangning Hospital, Ningbo, China; ^4^Central Laboratory of the Medical Research Center, Ningbo First Hospital, Ningbo Hospital of Zhejiang University, Ningbo, China

**Keywords:** Alzheimer’s disease, NMDAR, synaptic plasticity, cognitive behavior, dendritic morphology

## Abstract

The N-methyl-D-aspartate receptor is a critical molecule for synaptic plasticity and cognitive function. Impaired synaptic plasticity is thought to contribute to the cognitive impairment associated with Alzheimer’s disease (AD). However, the neuropathophysiological alterations of N-methyl-D-aspartate receptor (NMDAR) function and synaptic plasticity in hippocampal CA1 in transgenic rodent models of AD are still unclear. In the present study, APP/PS1 mice were utilized as a transgenic model of AD, which exhibited progressive cognitive impairment including defective working memory, recognition memory, and spatial memory starting at 6 months of age and more severe by 8 months of age. We found an impaired long-term potentiation (LTP) and reduced NMDAR-mediated spontaneous excitatory postsynaptic currents (sEPSCs) in the hippocampal CA1 of APP/PS1 mice with 8 months of age. Golgi staining revealed that dendrites of pyramidal neurons had shorter length, fewer intersections, and lower spine density in APP/PS1 mice compared to control mice. Further, the reduced expression levels of NMDAR subunits, PSD95 and SNAP25 were observed in the hippocampus of APP/PS1 mice. These results suggest that NMDAR dysfunction, impaired synaptic plasticity, and disrupted neuronal morphology constitute an important part of the neuropathophysiological alterations associated with cognitive impairment in APP/PS1 mice.

## Introduction

Alzheimer’s disease is a progressive neurologic disorder characterized by cognitive dysfunction, mainly learning and memory. The pathological alterations in the hippocampus have clinical consequences in cognitive impairment in Alzheimer’s disease (AD). The volume of the hippocampus was reduced in patients with AD, which can be used as an auxiliary examination to improve the accuracy of AD diagnosis (Hampel et al., 2002; Ewers et al., 2011). Particularly, the hippocampal CA1 area is one of the most influenced regions in AD (Gómez-Isla et al., 1997; Llorens-Martín et al., 2014; Yang et al., 2018), which is involved in spatial orientation, learning, and different aspects of memory, such as consolidation and retrieval (Bartsch et al., 2011; Fouquet et al., 2012). The impairment of these functions is related to the core clinical symptom in AD patients (Llorens-Martín et al., 2014). In animal models of AD, studies have found that abnormalities of neuronal morphology and expression levels of synapse-associated proteins contribute to the impaired neural plasticity in the hippocampal CA1 in AD models (Le Douce et al., 2020; Wang et al., 2020; Li et al., 2021).

N-methyl-D-aspartate receptors (NMDARs) are the major ligand-gated glutamate receptors and are crucial for neuronal development, synaptic plasticity, and excitotoxicity (Li et al., 2003). Amyloid beta (Aβ), one of the hallmarks of AD, directly disturbed NMDAR function in cultured neurons, whether synthetic or naturally secreted Aβ; in the cultured neurons from APPswe mice, the surface expression of NR1, an NMDAR subunit, was lower than neurons from WT mice (Snyder et al., 2005). Memantine, one of the commonly used drugs for AD, is the NMDAR antagonist and protects the neurons from excitatory toxicity (Parsons et al., 2007; Martinez-Coria et al., 2010). D-serine, as an NMDAR co-agonist, effectively prevents both synaptic and behavioral deficits in AD mice (Le Douce et al., 2020). In clinical researches, the blood level of D-amino acid oxidase was negatively correlated with age-related cognitive decline (Lin et al., 2017); benzoate, a D-amino acid oxidase inhibitor, substantially improved cognitive function in randomized, double-blind, placebo-controlled trials (Lin et al., 2014; Lane et al., 2021). These pieces of evidence imply that the changes of NMDAR function are complicated in AD, which may be attributable to the fact that AD is a heterogeneous disorder with various phenotypes and genotypes (Devi and Scheltens, 2018). The neuropathophysiological alterations of NMDAR function and synaptic plasticity in hippocampal CA1 in transgenic rodent models of AD are still unclear.

The transgenic mouse, APPswe/PS1dE9 (APP/PS1), is one of the most commonly used animal models in pathogenesis studies of AD (Hall and Roberson, 2012). Previous studies have reported that Aβ deposits and cognitive deficits were developed around 6–7 months of age in these mice (Jankowsky et al., 2004). In the present study, we assessed cognitive functions of APP/PS1 mice at 4, 6, and 8 months of age, and then investigated synaptic plasticity and NMDAR function in hippocampal CA1 *via* examining long-term potentiation (LTP) induction and NMDAR-mediated spontaneous excitatory postsynaptic currents (sEPSCs) in 8-month-old APP/PS1 mice. Furthermore, to understand the structural and molecular basis of altered synaptic plasticity, the neuronal morphology and expression levels of synapse-associated proteins, including glutamate receptor subunits, PSD95, and SNAP25, were determined using Golgi staining and western blotting, respectively. These results suggested that NMDAR dysfunction, impaired synaptic plasticity, and disrupted neuronal morphology constitute an important part of the neuropathophysiological alterations associated with cognitive impairment in APP/PS1 mice.

## Materials and Methods

### Animal

All experiments were conducted in line with the National Institutes of Health Guide for the Care and Use of Laboratory Animal, which were approved by the Animal Care and Use Committees of Ningbo University. APP/PS1 mice, purchased from the Hangzhou Ziyuan, Inc. of China, express a chimeric mouse/human APP gene harboring the Swedish double mutation K595N/M596L (APPswe) and a human PS1 gene harboring the exon 9 deletion (PS1dE9). The mice were housed under a 12 h light/dark cycle in groups of three to five per cage with free access to food and water. The breeding room was a temperature (20–22°C) and humidity (45–55%) controlled environment. Both female and male mice were used in the experiment and were equally distributed in each group.

### Behavioral Tests

All behavioral experiments were performed in three age stages (4, 6, and 8 months) between 7:00 a.m. and 6:00 p.m. The week before the behavioral test, each mouse was habituated to a room and a single experimenter, who handled subjects in the behavioral room for 5 min per day. The behavioral data were recorded and analyzed by ANY-maze software (Stoelting, United Kingdom).

#### Open Field Test

Open-field tests (OFTs) were conducted to test locomotor activity by a white box (40 cm long × 40 cm wide × 40 cm high) made of Plexiglas plate. Mice were placed in the center of the bottom of the box to explore freely for 10 min with a video camera recording their movements. The total distance traveled, the time spent in the central area (20 cm × 20 cm), and the immobile time (the timer started when the mouse was stationary for more than 2 s) of each mouse were measured.

#### Y-Maze

Y-maze tests were performed to assess short-term spatial working memory by spontaneous alternations. The device is a three-arm horizontal maze (40 cm long and 10 cm wide with 25 cm high) in which the angle between each of the two adjacent arms is 120°. Mice were placed at the center of the three arms and allowed to explore freely for 8 min. The total arm entries and sequences of each arm were recorded. The percent alternations, reflecting spatial working memory, were defined as the proportion of arm choices that differed from the last two choices.

#### Novel Location Recognition and Novel Object Recognition

Novel location recognition (NLR) and novel object recognition (NOR) tests were performed to test the ability of learning and memory in mice. In the NLR test, the device includes a small open field box (25 cm long × 25 cm wide × 25 cm high walls, one of whose walls are specially marked) and two identical objects. Mice were acclimated to the open field box for 3 days, 10 min a day. During training, 24 h after the last acclimatization, mice were allowed to explore two identical objects for 10 min. Investigation time for each object was measured. Afterward, 1 h after training for the test, one of the objects was picked up and placed to the opposite side of the box, and mice were allowed again to explore two objects for 5 min. The time of investigation for each object was measured again. Object exploration time was measured for each case in which the nose of a mouse touched the object or was oriented toward the object and came within 2 cm of it. The NLR discrimination index, reflecting spatial memory, was defined as (novel location investigation time - familiar location investigation time)/(novel location investigation time + familiar location investigation). NOR tests were performed as previously described, with minor modification. The device is the same as the NLR and two objects of different shapes but of the same material. Also, the acclimating and training phases are the same as the NLR test in the first 4 days. On the fifth day (24 h after the training phases), one of the objects was replaced with a new object, mice were allowed to explore two different objects for 5 min. The data are recorded in the same way as the NLR test. The NOR discrimination index, reflecting long-term recognition memory, was defined as (novel object investigation time – familiar object investigation time)/(novel object investigation time + familiar object investigation time).

#### Barnes Maze

The Barnes maze was performed to estimate spatial learning and memory. The maze consists of a white circular plate, 90 cm in diameter with 20 evenly distributed circular holes on the edge. The diameter of the holes is 5 cm and it is 4 cm from the plate edge. The maze was elevated above the floor (90 cm), light intensity was set at 200 lx, which was regarded as a mild aversive stimulus and visual cues were on the wall of the test room (within 50 cm of the maze). The test consists of 4 days of training and 1 day of probes. During training, under one of these holes is a dark box made of black Plexiglas with dimensions of 10 cm long × 10 cm wide × 6 cm high (escape box), where animals can stay here to some extent away from light and open spaces. Mice were trained to learn the location of the escape box over 4 days with 4 trials per day. At the beginning of each training session, mice were acclimated to the maze in the metal cylindrical cage at the center of the platform for 30 s. Next, mice were allowed to explore the maze to find and reach the escape box. The maximum duration of each training session was 180 s, and the interval between two adjacent training sessions was 15–25 min. If the mice fail to reach the escape box, the experimenter would gently guide it into the escape box by the end of 180 s. At the end of each training session (mice reached the escape box), every mouse was allowed to remain in the escape box for 30 s before returning to the home cage. On the fifth day, in the probe session, each mouse was allowed to explore the maze only for 90 s with the escape box removed. The maze was cleaned with 70% EtOH and rotated 90° after each session to avoid the effects of odors. The escape latency is the time from leaving the metal cylindrical cage to reaching the escape box. The target quadrant is the quadrant in which the escape hole was on its axis of symmetry. Errors mean visits to incorrect holes before finding the escape hole.

### Electrophysiology

The electrophysiology was performed as previously described (Kasanetz and Manzoni, 2009; Zhang et al., 2021). Coronal brain slices containing hippocampal CA1 were prepared for field excitatory postsynaptic potentials (fEPSPs) and whole-cell recording from WT and APP/PS1 mice during PND 250–270. Mice were anesthetized with sodium pentobarbital (80 mg/kg, i.p.) and decapitated, and then brains were dissected quickly and placed in an ice-cold solution containing below substances (in mM): 75 sucrose, 87 NaCl, 3.0 KCl, 1.5 CaCl_2_, 1.3 MgCl_2_, 1.0 NaH_2_PO_4_, 26 NaHCO_3_, 20 glucose equilibrated with 95% O_2_–5% CO_2_. Coronal brain slices (220 μm thickness) were prepared with a vibratome (Leica VT1200S, Leica Microsystems, Germany), and then incubated in a chamber with artificial cerebrospinal fluid (aCSF) containing below substances (in mM): 124 NaCl, 3.0 KCl, 1.0 NaH_2_PO_4_, 1.3 MgCl_2_, 2.0 CaCl_2_, 26 NaHCO_3_, and 20 glucose, 295–305 mOsm, equilibrated at 32°C with 95% O_2_–5% CO_2_). Slices were incubated for at least 1 h before recording. Following incubation, the slices were transferred to a recording chamber, where the submerged slices were perfused with aCSF (32°C) saturated with mixed gas at a flow rate of 2 ml per min. Standard recordings were made using Multiclamp 700B amplifier and Digidata 1550B (Molecular Devices, Axon Instruments, CA, United States) for data acquisition. Vertical two stages puller (PC-10, NARISHIGE, JAPAN) was used to make glass electrodes (3IN thin-wall GL1.5 OD/1.12 ID, TW150-3, WPI) into pipettes with resistance between 1.5 and 2.0 mOhm when filled with internal. The fEPSPs were recorded with glass electrodes (∼3 MΩ tip resistance) filled with ACSF and evoked with a bipolar tungsten electrode (FHC, Inc., United States). Stimulus strength was adjusted to ∼40% of the maximal fEPSP response. For LTP recording, after a 20 min stable baseline was established, LTP was induced by theta-burst stimulation (TBS) (four theta bursts were applied at 15 s intervals; each theta-burst consisted of five bursts, at 200 ms intervals, of five 100 Hz pulses). For whole-cell recording, to record NMDAR-mediated sEPSCs, patch pipettes were filled with an intracellular solution containing (in mM): 110 cesium methyl sulfate, 15 CsCl, 4.0 Mg-ATP, 0.3 Na2-GTP, 0.5 EGTA, 10 HEPES, 4.0 QX-314, 5.0 Phosphocreatine-Na2, pH 7.2–7.4 (270–280 mOsm). sEPSCs mediated by both AMPAR and NMDAR were detected with digitally designed templates (Molecular Devices). The sEPSC charge was computed by the following formula: sEPSC charge = current (pA) × time (ms). For dual sEPSCs, a template with the rise and decay times of 3 and 150 ms, respectively, was used. A lower-amplitude threshold of 16 pA was applied. AMPAR sEPSCs at +40 mV were isolated in the presence of the NMDAR antagonist D-2-amino-5-phosphonovaleric acid (D-APV, 14539, Cayman, United States; 100 μM) with a template with the rise and decay times of 1.2 and 4 ms, respectively. A lower-amplitude threshold of 9 pA was applied. The NMDAR sEPSCs were obtained by the following formula: NMDAR sEPSCs = dual (AMPAR + NMDAR) sEPSCs – AMPAR sEPSCs. Series resistance was normally <20 MΩ and recordings exceeding 20% change in series resistance were terminated. All holding potentials were corrected for liquid junction potential. Data were low-pass filtered at 1 kHz and digitized at a sampling frequency of 10 kHz. The superfusion medium contained picrotoxin (P1675, Sigma, Germany; 100 μM) to block γ-aminobutyric acid type A receptors. All chemicals used in the patch-clamp were purchased from Sinopharm Chemical Reagent Co., Ltd., China, except as noted.

### Golgi-Cox Staining and the Analysis of Dendrites and Spines

Mice brains were immersed in the Golgi-Cox solution for 2 weeks and then transferred to 30% sucrose solution for 3–5 days in the dark at RT (room temperature). Coronal slices (140 μm thickness) containing the hippocampus were cut by a vibratome (Leica VT1200S, Leica Microsystems, Germany). Pyramidal neurons in the hippocampal CA1 region were selected for structural analysis. Total dendritic length and the number of intersections at concentric circles (10 μm apart) were measured by Sholl analysis, and the number of dendritic spines per 10 μm was analyzed using ImageJ software (version 1.52a; National Institutes of Health).

### Immunofluorescent Staining and Quantitative Analysis

Brains were isolated and fixed in 4% paraformaldehyde (PFA) for 24 h and then cryoprotected in 30% sucrose solution in PBS 1X for additional 2 days. After fixation and cryoprotection procedures, brains were cut using Leica Cryostat in 30 μm thickness. Sections were transferred into a blocking solution containing 0.1% Triton X-100, 10% goat serum in PBS 1X for 1 h at RT. Then, sections were incubated at 4°C overnight with the primary antibody, rabbit anti-NeuN (26975-1-AP; Proteintech, Wuhan, China; 1:500) diluted in PBS 1X, 0.1% Triton X-100, and 10% goat serum. After washing with PBS 1X for 1 h, the sections were incubated with secondary antibodies (Proteintech, Wuhan, China) diluted in PBS 1X for 2 h at RT. Afterward, sections were washed by PBS 1X thoroughly for 1 h and mounted with an antifading medium (Solarbio, Beijing, China). For quantitative analysis of NeuN staining, the average fluorescence intensity was determined using ImageJ software (version 1.52a; National Institutes of Health).

### Western Blot Analysis

The mice were deeply anesthetized with pentobarbital (80 mg/kg, i.p.) and decapitated during PND 250-270. Briefly, the hippocampus tissues were directly lysed in sodium dodecyl sulfate sample buffer and incubated at 95°C for 5 min before being loaded onto a 10% sodium dodecyl sulfate-polyacrylamide gel. The protein concentration was determined before being loaded onto the gel and then 40 μg protein per sample was loaded onto each track. Proteins were separated by electrophoresis and then transferred to a nitrocellulose membrane (BioRad, Hercules, CA, United States). The membrane was blocked in 5% milk-TBST at RT and probed with rabbit anti-NR2A (ab133265, Abcam, United Kingdom; 1:1,000), rabbit anti-NR2B (ab183942, Abcam, United Kingdom; 1:1,000), rabbit anti-NR1 (ab109182, Abcam, United Kingdom; 1:1,000), rabbit anti-GluR1 (04-855, Millipore, Germany; 1:1,000), mouse anti-GluR2 (MAB397, Millipore, Germany; 1:1,000), PSD95 antibody (sc2290, Santa Cruz Biotechnology Inc., United States; 1:500), SNAP25 antibody (A2234, Abclonal, China; 1:500) at 4°C overnight and then reacted with the Alexa Fluor 800 conjugated antibody (1:5,000) for 60 min. Detection and quantification of specific bands were performed using a fluorescence scanner (Odyssey Infrared Imaging System, LI-COR Biotechnology, Lincoln, NE, United States). All samples were analyzed at least in triplicate. β-Actin (AC026, Abclonal, China; 1:5,000) served as an internal protein control.

### Statistical Analysis

GraphPad Prism version 7 (GraphPad Software, San Diego, CA, United States) was used to conduct statistical analyses. Unpaired Student’s *t*-test was used to compare pairs of means. Two-way ANOVA followed by Bonferroni’s *post hoc* test when appropriate was used to analyze the latency to escape in the Barnes maze. The linear relationship between mobility and cognitive performance was analyzed by the Pearson’s coefficient after a Shapiro-Wilk normality test. Two-way repeated-measures ANOVA by Bonferroni’s *post hoc* test when appropriate was used to analyze the number of dendritic intersections. Data are presented as mean ± SEM. *p* < 0.05 was considered statistically significant for all results.

## Results

### Impaired Cognitive Function in 6- and 8-Month-Old APP/PS1 Mice

**FIGURE 1:**
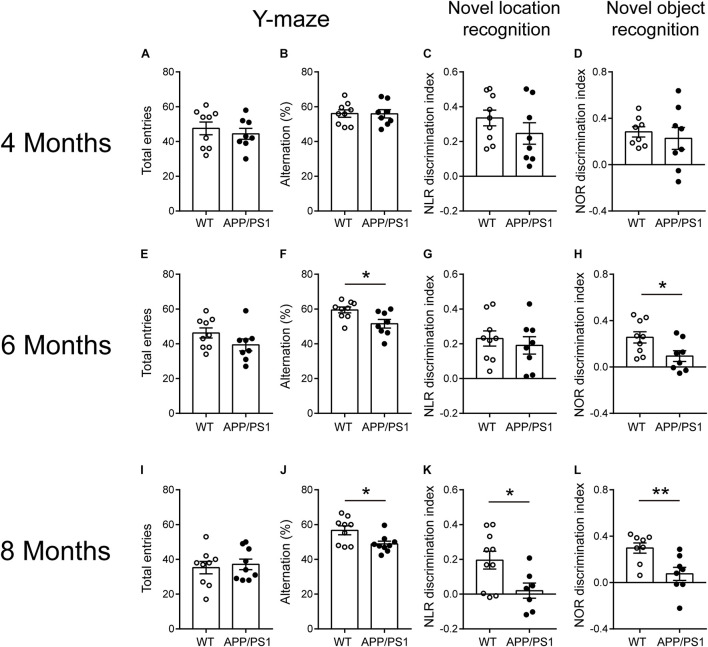
Cognitive-behavioral performance for 4-, 6-, and 8- months-old WT and APP/PS1 mice (*n* = 8–10/group). **(A–D)** 4-month-old WT and APP/PS1 mice showed no difference in Y-maze, NLR, and NOR tests. **(E–H)** In 6-month-old mice, APP/PS1 mice showed the decreased spontaneous alternations in Y-maze (*p < 0.05, unpaired *t*-test), reduced discrimination index in NOR (*p < 0.05, unpaired *t*-test) compared with WT mice, and no differences were observed in the number of total arm entries in Y-maze and discrimination index in NLR. **(I–L)** In 8-month-old mice, APP/PS1 mice showed the decreased spontaneous alternations in Y-maze (*p < 0.05, unpaired *t*-test), the reduced (*p < 0.05, unpaired *t*-test) discrimination index in NOR and NLR tests (**p < 0.001, unpaired *t*-test) compared with WT mice and there was no difference on the number of total arm entries in Y-maze.

The behavioral tests including Y-maze, NLR, and NOR are easy-to-use to measure different aspects of cognitive function. Four-month-old APP/PS1 mice showed no difference from age-matched WT mice in Y-maze [Figures 1A,B; total entries, unpaired *t*-test: *t*_(15)_ = 0.6515; *P* = 0.5246; alternations, unpaired *t*-test: *t*_(15)_ = 0.0382; *P* = 0.97], NLR [Figure 1C; unpaired *t*-test: *t*_(15)_ = 1.182; *P* = 0.2555], and NOR tests [Figure 1D; unpaired *t*-test: *t*_(14)_ = 0.5405; *P* = 0.5973]. The 6-month-old APP/PS1 mice exhibited the decreased spontaneous alternations in Y-maze [Figure 1F; unpaired *t*-test: *t*_(15)_ = 2.692; *P* = 0.0167], the reduced discrimination index in NOR [Figure 1H; unpaired *t*-test: *t*_(15)_ = 2.402; *P* = 0.0297] compared with WT mice. No differences were observed in the number of total arm entries in Y-maze [Figure 1E; unpaired *t*-test: *t*_(15)_ = 1.534; *P* = 0.1458] and the discrimination index in NLR [Figure 1G; unpaired *t*-test: *t*_(15)_ = 0.5997; *P* = 0.5577]. The 8-month-old APP/PS1 mice showed the decreased spontaneous alternations in Y-maze [Figure 1J; unpaired *t*-test: *t*_(16)_ = 2.585; *P* = 0.0199], the reduced discrimination index in NOR and NLR tests [Figures 1K,L; NLR, unpaired *t*-test: *t*_(15)_ = 2.464; *P* = 0.0263; NOR, unpaired *t*-test: *t*_(14)_ = 3.097; *P* = 0.0079] compared with WT mice, and no difference in the number of total arm entries in Y-maze [Figure 1I; unpaired *t*-test: *t*_(16)_ = 0.4037; *P* = 0.6918]. These results suggested that APP/PS1 mice manifested impaired working memory and long-term recognition memory at 6 months of age, and the impaired spatial recognition memory at 8 months of age.

### Impaired Spatial Learning and Memory in 6- and 8-Month-Old APP/PS1 Mice

**FIGURE 2:**
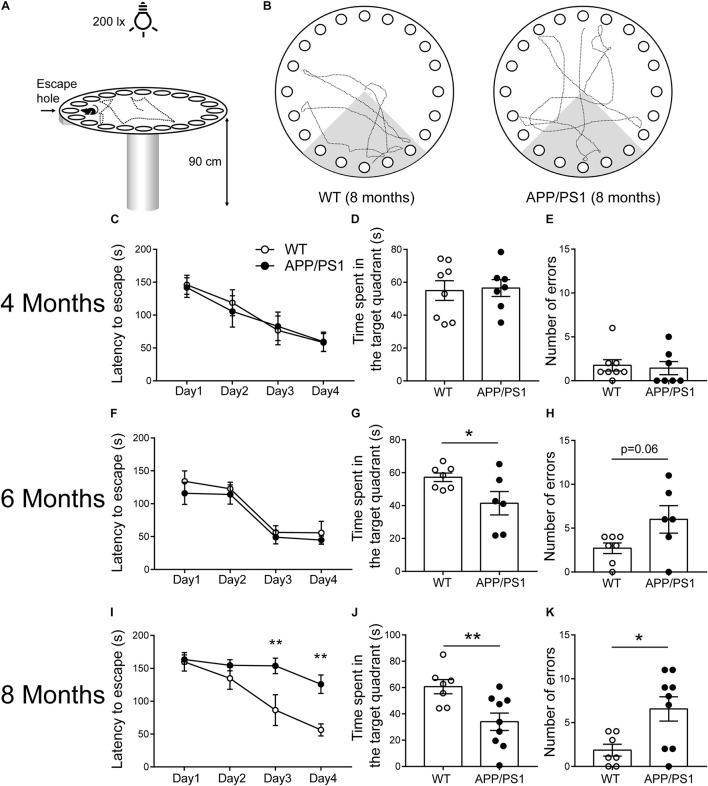
Barnes maze performance for 4-, 6-, and 8-months-old WT and APP/PS1 mice (*n* = 6–9/group). **(A)** The illustration shows our Barnes maze installation. **(B)** Example of trajectories of 8-month-old WT and APP/PS1 mice during the probe session. **(C–E)** In 4-month-old WT and APP/PS1 mice, no statistically significant differences were observed in the indicators (latency to escape, time spent in the quadrant, and the number of errors) of the Barnes maze. **(F–H)** In 6-month-old mice, the time APP/PS1 mice spent in the target quadrant is decreased compared with WT mice (*p < 0.05, unpaired *t*-test), but no differences were observed in the escape latency and the number of errors. **(I–K)** In 8-month-old APP/PS mice, the latency to escape was decreased in both day 3 and 4 of the training phase (interaction: *p < 0.05; at Day 3 or Day 4, **p < 0.01, two-way ANOVA followed by Bonferroni’s *post hoc* test), the time spent in the target quadrant was reduced (**p < 0.01, unpaired *t*-test), and the number of errors was increased compared with WT mice (*p < 0.05, unpaired *t*-test).

We found that APP/PS1 mice showed abnormalities in some aspects of cognitive function at 6 months of age, and more comprehensive cognitive impairment at 8 months of age through some simple behavioral tests. As a more complex behavioral task, the Barnes maze test (Figure 2A) was conducted to further verify cognitive dysfunction in APP/PS1 mice. In 4-month-old WT and APP/PS1 mice, no statistically significant differences were observed in the indicators of the Barnes maze (Figures 2C–E). In 6-month-old mice, the time APP/PS1 mice spent in the target quadrant is decreased [Figure 2G; unpaired *t*-test: *t*_(11)_ = 2.216; *P* = 0.0487] compared with WT mice, but no differences were observed in the escape latency [Figure 2F; two-way ANOVA: factor of day: *F*_(3, 44)_ = 18.31, *P* < 0.0001; factor of genotype: *F*_(1, 44)_ = 1.425, *P* = 0.2389; interaction of two factors: *F*_(3, 44)_ = 0.066, *P* = 0.9776] and the number of errors [Figure 2H; Unpaired *t*-test: *t*_(11)_ = 2.071; *P* = 0.0626]. The Figure 2B showed the trajectory diagrams of 8-month-old APP/PS1 mice, the escape latency was decreased in both day 3 and 4 of training phase [Figure 2I; two-way ANOVA followed by Bonferroni’s *post hoc* test: factor of day: *F*_(3, 56)_ = 18.31, *P* < 0.0001; factor of genotype: *F*_(1, 56)_ = 17.92, *P* < 0.0001; interaction of two factors: *F*_(3, 56)_ = 3.114, *P* = 0.0333], the time spent in the target quadrant was reduced [Figure 2J; unpaired *t*-test: *t*_(14)_ = 2.986; *P* = 0.0098], and the number of errors was increased compared with WT mice [Figure 2K; unpaired *t*-test: *t*_(14)_ = 2.783; *P* = 0.0147]. These results were consistent with the findings in the NLR test, suggesting that APP/PS1 mice have a progressive decline of memory task performance, which is closely related to the hippocampus as well.

### Reduced Locomotor Activity in APP/PS1 Mice at the Age of 6 Months

**FIGURE 3:**
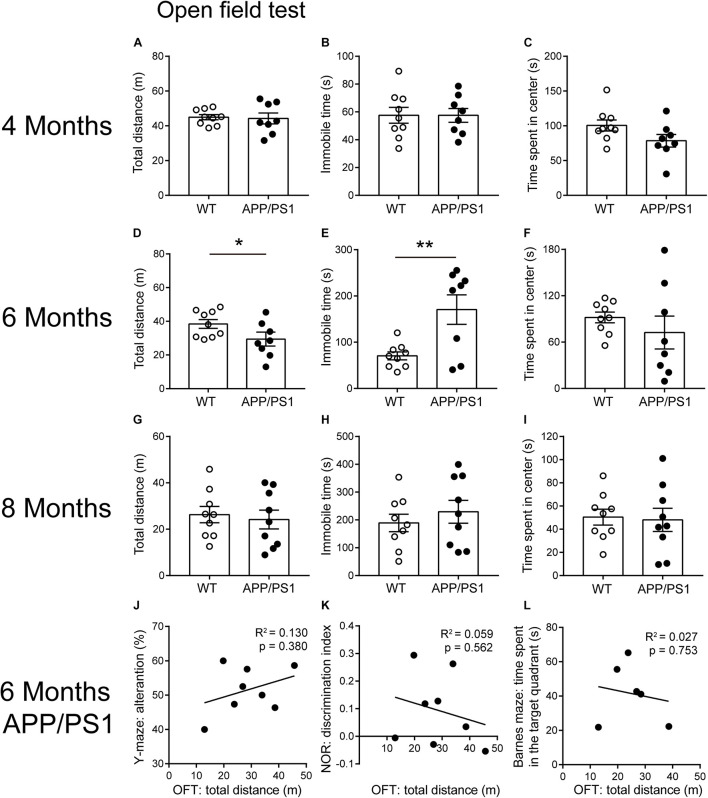
Open field test performance for 4-, 6-, and 8-month-old WT and APP/PS1 mice (*n* = 8–9/group). **(A–C)** In 4-month-old WT and APP/PS1 mice, no statistically significant differences were observed in the indicators (total distance, time spent in the center, and immobile time) of OFT. **(D–F)** In 6-month-old mice, APP/PS1 mice showed reduced total distance (*p < 0.05, unpaired *t*-test) and increased immobile time (**p < 0.001, unpaired *t*-test) in OFT, compared with WT mice, but there was no difference in the time spent in the center. **(G–I)** In 8-month-old mice, there were no differences in these indicators of OFT. **(J–L)** There was no significant correlation between the total distance in OFTs and the performance in Y-maze, NOR, or Barnes maze in 6-month-old APP/PS1 mice. Each point on these graphs represents an independent mouse.

Open field test is a basic measurement of exploratory and locomotor activity in rodents. Our results showed that the total distance was decreased only in the 6-month-old APP/PS1 mice, compared with age-matched WT mice [Figure 3D; unpaired *t*-test: *t*_(15)_ = 2.166; *P* = 0.0469]. Accordingly, the 6-month-old APP/PS1 mice showed significantly increased periods of immobility in OFTs [Figure 3E; unpaired *t*-test: *t*_(15)_ = 3.194; *P* = 0.006]. However, when 4- and 8-month-old mice were used as experimental subjects, no statistically significant differences were observed in the above two indicators between APP/PS1 mice and WT mice (Figures 3A,B,G,H). Concerning the time spent in the center, there were no differences between APP/PS1 mice and WT mice at 4, 6, and 8 months of age, respectively (Figures 3C,F,I). These results suggested that 6-month-old APP/PS1 mice showed a decreased locomotor activity in the novel environment. To evaluate whether the magnitude of mobility loss is sufficient to cause impairment in Barnes maze (Figure 2G), Y-maze (Figure 1F), and NOR (Figure 1H) in the 6-month-old APP/PS1 mice, we analyzed the correlation between the total distance of OFTs and performance in Y-maze (Figure 3J), NOR (Figure 3K), or Barnes maze (Figure 3L). However, no significant correlation between the mobility and cognitive performance was found in 6-month-old APP/PS1 mice (Figure 3J, *R*^2^ = 0.13, *P* = 0.38; Figure 3K, *R*^2^ = 0.059, *P* = 0.562; Figure 3L, *R*^2^ = 0.027, *P* = 0.753).

### Impaired Long-Term Potentiation in the CA1 of 8-Month-Old APP/PS1 Mice

**FIGURE 4:**
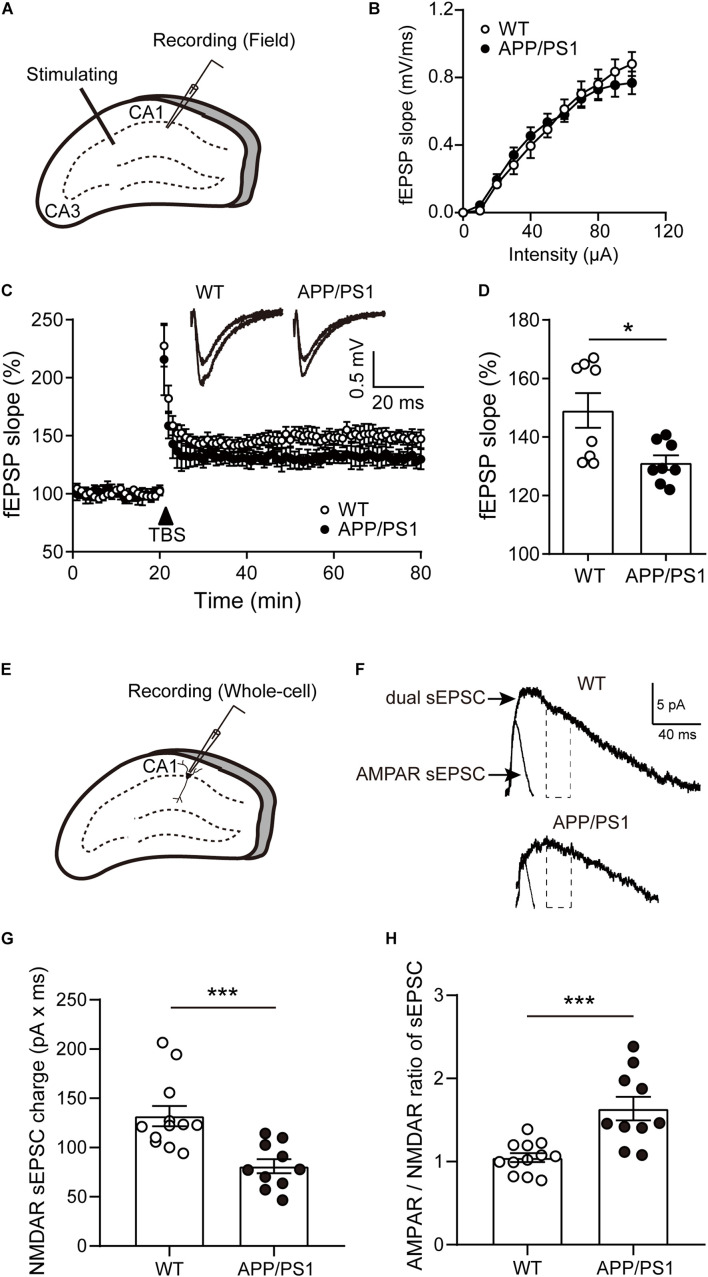
The LTP and NMDAR sEPSCs of pyramidal neurons in the CA1 of 8-month-old APP/PS1 mice. **(A)** Schematic diagram of fEPSP recording. **(B)** The input-output (I/O) curves in response to stimulus in the CA1 of WT and APP/PS1 mice. **(C)** Representative traces showing LTP in CA1 of WT and APP/PS1 mice. **(D)** Quantification of the last 15 min of fEPSP recordings (*n* = 8 slices from 4 mice/group; *p < 0.05, unpaired *t*-test). **(E)** Schematic diagram of whole-cell recording. **(F)** The diagram showed typical average events from WT and APP/PS1 mice. NMDAR sEPSCs were estimated from the charge of the dual sEPSCs after AMPAR contribution decayed to zero. Dotted lines represent the time window in which the NMDAR sEPSCs charge was calculated. **(G)** The NMDAR sEPSCs charge was reduced in the cells from APP/PS1 (*n* = 10–12 cells from 4 to 6 mice/group; ***p < 0.001, unpaired *t*-test). **(H)** The AMPAR/NMDAR ratio of sEPSCs was increased in pyramidal neurons of CA1 from APP/PS1 mice, compared with WT mice (***p < 0.001, unpaired *t*-test). contribution decayed to zero. Dotted lines represent the time window in which the NMDAR sEPSCs charge was calculated. **(G)** The NMDAR sEPSCs charge was reduced in the cells from APP/PS1 (*n* = 10–12 cells from 4 to 6 mice/group; ***p < 0.001, unpaired *t*-test). **(H)** The AMPAR/NMDAR ratio of sEPSCs was increased in pyramidal neurons of CA1 from APP/PS1 mice, compared with WT mice (***p < 0.001, unpaired *t*-test).

Our behavioral results found that impaired hippocampal-dependent cognition in 8-month-old APP/PS1 mice. We then examined the LTP at the Schaffer collateral-CA1 pyramidal neuron synapses of APP/PS1 mice at the same age (Figure 4A). First, the dependence of the fEPSP on stimulation intensity was analyzed in input/output curves [Figure 4B; two-way repeated-measures ANOVA: factor of genotype: *F*_(1, 3)_ = 0.01896, *P* = 0.8992; factor of intensity: *F*_(10, 30)_ = 168.9, *P* < 0.0001; interaction of two factors: *F*_(10, 30)_ = 0.2026]. This result showed that the baseline synaptic transmission was not changed in 8-month-old APP/PS1 mice compared with the age-matched WT mice. Next, LTP was induced by TBS after 20 min of stable baseline recording (Figure 4C). Our results revealed that LTP in CA1 area of 8-month-old APP/PS1 mice was significantly depressed in comparison with control mice [Figure 4D; WT: 149.1 ± 5.935, APP/PS1: 131.2 ± 2.48, unpaired *t*-test: *t*_(14)_ = 2.772; *P* = 0.015], reflecting the deficits in synaptic plasticity.

### Reduced Spontaneous N-Methyl-D-Aspartate Receptor Current of Pyramidal Neurons in the CA1 of 8-Month-Old APP/PS1 Mice

Furthermore, we recorded NMDAR-mediated spontaneous excitatory postsynaptic currents (sEPSCs) *via* whole-cell recording (Figure 4E). When presumed pyramidal neurons were held at +40 mV, sEPSCs consisted of both α-amino-3-hydroxy-5-methyl-4-isoxazole propionic acid receptor (AMPAR)- and NMDAR-mediated current (dual sEPSCs). Within 30 ms after dual sEPSCs appeared, a time point at which AMPAR sEPSCs charge already decayed to zero. Next, AMPAR sEPSCs were isolated by the presence of D-APV (Figure 4F). The results showed that NMDAR sEPSCs were largely diminished in 8-month-old APP/PS1 mice [Figure 4G; unpaired *t*-test: *t*_(20)_ = 3.885; *P* = 0.0009]. In addition, the ratio of the AMPAR sEPSCs to NMDAR sEPSCs was significantly increased in pyramidal neurons from APP/PS1 mice [Figure 4H; unpaired *t*-test: *t*_(20)_ = 4.192; *P* = 0.0004]. Taken together, these data suggest the functional decline of NMDAR in APP/PS1 mice.

### Reduced Morphological Complexity and Dendritic Spines of Pyramidal Neurons in 8-Month-Old APP/PS1 Mice

**FIGURE 5:**
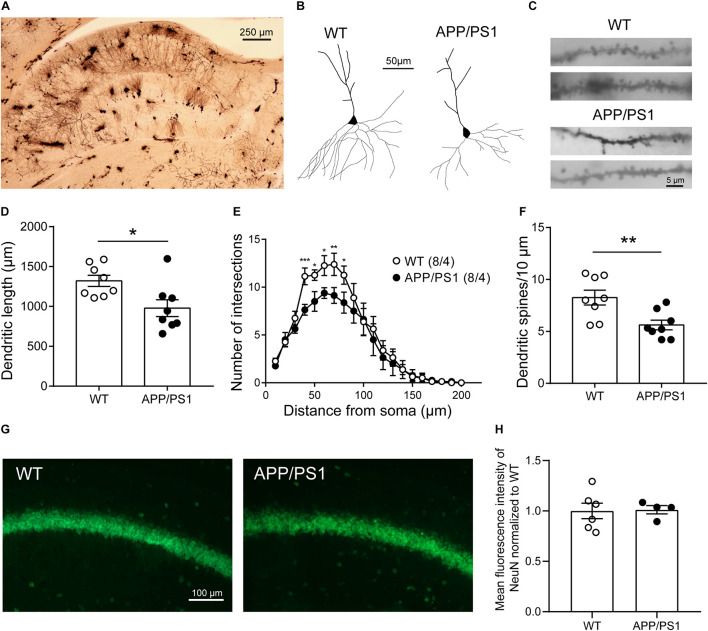
The morphology and number of pyramidal neurons in the CA1 of 8-month-old APP/PS1 mice. **(A)** Golgi-Cox staining of brain tissue samples. Scale bar, 250 μm. **(B)** Representative examples of reconstructed pyramidal neurons in the CA1 of 8-month-old WT and APP/PS1 mice. Scale bar, 50 μm. **(C)** Representative spine morphology of pyramidal neurons in WT and APP/PS1 mice. Scale bar, 5 μm. **(D)** The dendritic length of pyramidal neurons was decreased in APP/PS1 mice compared with WT mice (*n* = 8 from 4 mice; *p < 0.05, unpaired *t*-test). **(E)** The complexity of dendrites of pyramidal neurons was decreased in APP/PS1 mice compared with WT mice (interaction: ***p < 0.001; two-way repeated-measures ANOVA followed by Bonferroni’s *post hoc* test, *p < 0.05, **p < 0.01, ***p < 0.001). **(F)** The total dendritic spine density of pyramidal neurons was decreased in APP/PS1 mice compared with WT mice (*n* = 8 from 4 mice; **p < 0.01, unpaired *t*-test). **(G)** Representative images of NeuN staining of the hippocampal CA1 area of 8-month-old APP/PS1 and WT mice. Scale bar: 100 μm. **(H)** The quantitative results of the mean fluorescence intensity of NeuN were not altered significantly between 8-month-old APP/PS1 and WT mice (*n* = 4–6 mice/group).

The reason behind attenuated LTP may also be abnormal neuronal morphology and changed synaptic density in the CA1 area. Then we performed Golgi-Cox staining to detect the morphology and dendritic spine density of pyramidal neurons in CA1 (Figure 5A). Anatomical reconstruction of analyzed pyramidal neurons (Figure 5B) showed that the dendritic length [Figure 5D; WT: 1,321 ± 69.72, APP/PS1: 979 ± 105.7, unpaired *t*-test: *t*_(14)_ = 2.702; *P* = 0.0172], and the number of intersections [Figure 5E; two-way repeated-measures ANOVA followed by Bonferroni’s *post hoc* test: factor of genotype: *F*_(19, 133)_ = 39.04, *P* < 0.0001; factor of distance from soma: *F*_(1, 7)_ = 2.727, *P* = 0.1427; interaction of two factors: *F*_(19, 133)_ = 0.001] were decreased in 8-month-old APP/PS1 mice, suggesting a lower level of complexity of cells. In addition, 8-month-old APP/PS1 mice exhibited a significantly lower spine density compared with WT mice [Figures 5C,F; WT: 8.258 ± 0.7125, APP/PS1: 5.617 ± 0.4642, unpaired *t*-test: *t*_(14)_ = 3.106; *P* = 0.0077]. Since the loss of neurons mainly in the hippocampus and cortex is another hallmark at this stage of AD (Niikura et al., 2006; Wirths and Zampar, 2020), we then conducted the fluorescence stainings of the NeuN antibody (Figure 5G). And no significant differences were observed between APP/PS1 and WT mice at 8 months of age [Figure 5H; unpaired *t*-test: *t*_(8)_ = 0.1188; *P* = 0.9084].

### Decreased Expression Levels of N-Methyl-D-Aspartate Receptor Subunits and Synapse-Associated Proteins in the Hippocampus of 8-Month-Old APP/PS1 Mice

**FIGURE 6:**
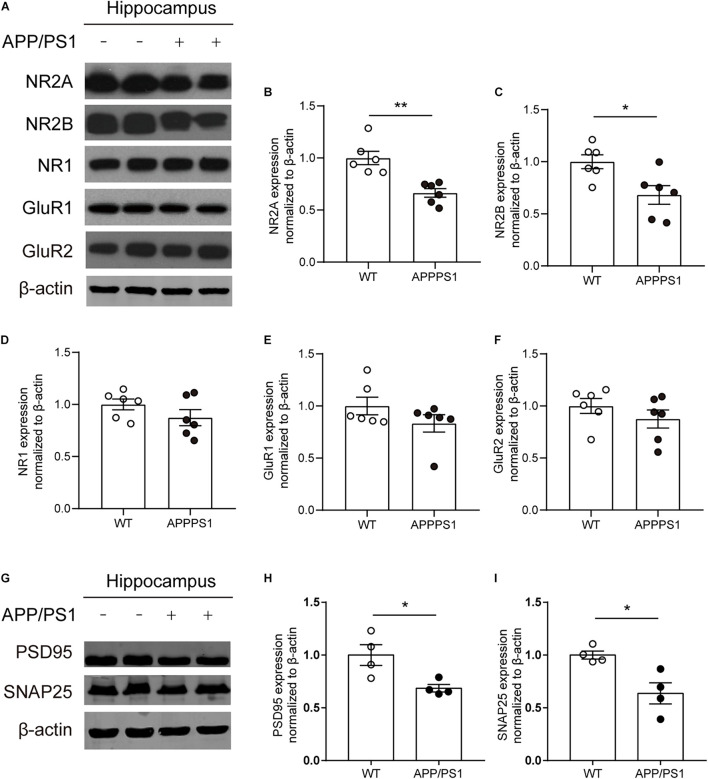
The expression of synapse-associated proteins and glutamate receptor subunit proteins in the CA1 area of 8-month-old WT and APP/PS1 mice. **(A)** Representative western blots of glutamate receptor subunits. **(B–D)** Quantitative of NMDAR subunits expression showed a significant decrease of NR2A and NR2B expression (*n* = 6 mice/group, *p < 0.05, **p < 0.01, unpaired *t*-test) in the hippocampus in 8-month-old APP/PS1 mice compared with WT mice, but there was no difference on the expression of NR1 from two groups. **(E,F)** Quantitative of AMPA receptors subunits (GluR1, GluR2) expression showed no change in hippocampus from 8-month-old APP/PS1 mice and WT mice. **(G)** Representative western blots of PSD95 and SNAP25. **(H,I)** A significant decrease of the levels of PSD95 and SNAP25 in the hippocampus from 8-month-old APP/PS1 mice compared with WT mice (*n* = 4 mice/group, *p < 0.05, unpaired *t*-test).

Combined with the above results, to determine whether functional alterations of NMDARs are caused by receptor subunits, the expression levels of NMDAR subunits NR1, NR2A, and NR2B and AMPAR subunits GluR1 and GluR2 in the hippocampus of 8-month-old APP/PS1 mice were detected (Figure 6A). The results showed that the expression levels of NR2A, NR2B significantly declined in the hippocampus of APP/PS1 mice [Figures 6B,C; NR2A, unpaired *t*-test: *t*_(10)_ = 4.364; *P* = 0.0014; NR2B, unpaired *t*-test: *t*_(10)_ = 2.853; *P* = 0.0172]. However, no statistically significant differences were observed in the expression levels of NR1, GluR1, and GluR2 between APP/PS1 mice and WT mice (Figures 6D–F). In addition, the expression levels of other synaptic function proteins were also detected (Figure 6G). The results showed that a significant decrease in the levels of postsynaptic density 95 (PSD95) [Figure 6H; unpaired *t*-test: *t*_(6)_ = 3.012; *P* = 0.0236] and synaptosomal-associated protein 25 (SNAP25) [Figure 6I; unpaired *t*-test: *t*_(6)_ = 3.41; *P* = 0.0143] was found in 8-month-old APP/PS1 mice.

## Discussion

**FIGURE 7:**
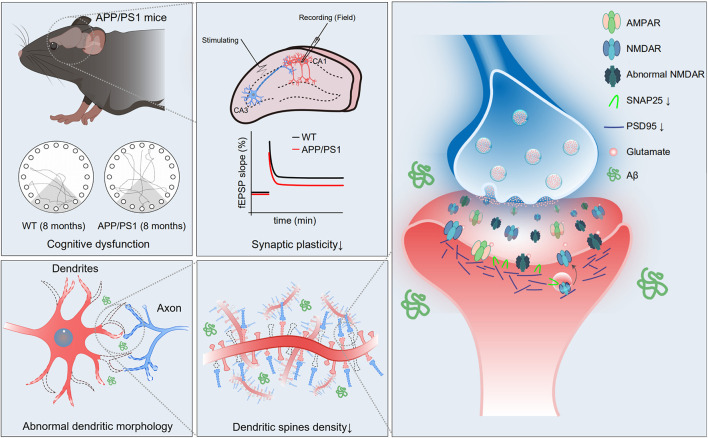
Schematic diagram of the mechanism of cognitive impairment in 8-month-old APP/PS1 mice. In hippocampal CA1 area of APP/PS1 mice, impaired function of NMDAR, reduced expression of synaptic proteins, abnormal neuronal morphology, and decreased dendritic spine density may give rise to weak synaptic plasticity, which mediates age-related cognitive dysfunction.

In the present study, we investigated synaptic plasticity and NMDAR function in hippocampal CA1 in 8-month-old APP/PS1 mice, because the cognitive dysfunction is more severe at this age. The results showed that LTP and NMDAR-mediated sEPSCs were reduced and pyramidal neuron dendrites had shorter length, fewer intersections, and lower spine density in the hippocampal CA1 of APP/PS1 mice. Further, the reduced expression levels of NMDAR subunits, PSD95 and SNAP25 were observed in the hippocampus of APP/PS1 mice. These results suggest that NMDAR dysfunction, impaired synaptic plasticity, and disrupted neuronal morphology constitute an important part of the neuropathophysiological alterations associated with cognitive impairment in APP/PS1 mice (Figure 7).

Cognitive dysfunction is the most significant symptom of AD, which is often preceded by a period of mild cognitive impairment (MCI). About one-third of all patients with MCI progress to AD in 5 years (Palmer et al., 2007). Consistent with previous reports (Bruce-Keller et al., 2011; Chen et al., 2012), we confirmed the deterioration of cognitive function with the increase of the age of APP/PS1 mice, which included working memory, short-term spatial memory, long-term recognition memory, and spatial learning and memory. These cognitive tasks mainly reflect the ability of hippocampus-dependent learning and memory (Zheng et al., 2009; Gonzalez et al., 2021), and the results hinted at an abnormality of hippocampal function in APP/PS mice at 8 months of age. To exclude the effect of locomotor activity in cognitive tests, we performed OFTs and found that APP/PS1 mice showed decreased locomotor activity in the novel environment at 6 months, but not at 8 months of age. However, correlation analysis showed that mobility loss was not associated with impaired cognitive performance in 6-month-old APP/PS1 mice. Interestingly, a decrease in locomotor activity was also reported in other AD models (Samaey et al., 2019) and aging mice (Shoji et al., 2016; Shoji and Miyakawa, 2019), the latter may suggest that reduced locomotor activity may be an indicator of aging. Taken together, genotype-specific pathogenic effects may accelerate aging and cause reduced mobility in 6-month-old APP/PS1 mice. By contrast, at 8 months of age, since the mobility of WT mice was also reduced due to aging, no significant differences were observed between the APP/PS1 and WT mice.

Long-term potentiation as an indicator of synaptic plasticity in the hippocampal CA1 area is deeply involved in spatial learning and memory (Bartsch et al., 2011; Fouquet et al., 2012). Reduced LTP of the CA1 area is often seen in diseases characterized or accompanied by cognitive dysfunction, such as AD, schizophrenia, and depression (Liu et al., 2019; Le Douce et al., 2020; Percelay et al., 2021). The mechanism of impaired synaptic plasticity in AD has been not fully understood. The elicitation of many forms of LTP requires NMDAR activation and the weakening or strengthening of the plasticity is controlled mainly by the kinetics of CA^2+^ influx through NMDARs (Yashiro and Philpot, 2008). Previous studies have found an impaired NMDAR function might be related to the pathophysiology of AD (Mota et al., 2014; Zhang et al., 2016; Wang and Reddy, 2017). For example, Aβ recruited internalization of synaptic NMDARs and the reduction of NMDAR-mediated currents in the cultured cortical neurons (Snyder et al., 2005). In addition, some novel therapies modulating NMDAR function have been shown to restore cognitive function in clinical trials (Goetghebeur et al., 2019; Lin et al., 2021). Taken together, there is growing evidence of NMDAR-associated synaptic abnormalities in AD, which is consistent with our results that NMDAR-mediated sEPSCs were reduced in hippocampal CA1 with impaired synaptic plasticity in APP/PS1 mice.

The structural adaptation of neurons is also an important form of synaptic plasticity. The loss of neurons, abnormal neuronal morphology like reduced dendritic spine density, and abnormal synaptic structure were all constantly associated with impaired LTP (Bencsik et al., 2019; Reza-Zaldivar et al., 2020; Meftahi et al., 2021). We found aberrant dendritic length, complexity, and decreased dendritic spine density of pyramidal neurons in the CA1 area of APP/PS1 mice. However, no change was observed in the number of neurons. These results suggested that pyramidal neurons in CA1 in AD mice might have lower efficiency of signal transmission with other neurons, which could be partly responsible for the impaired synaptic plasticity and cognition in APP/PS1 mice (Yuste and Bonhoeffer, 2001; Yang et al., 2015).

Experiments with western blots showed reduced expressing levels of NR2A and NR2B in hippocampal CA1 of APP/PS1 mice. These results support our electrophysiological findings, suggesting that the reduction of NMDAR-mediated sEPSCs may be due to the low expression of specific receptor subunits. In addition, decreased expression levels of PSD95 and SNAP25 were observed in hippocampal CA1 of APP/PS1 mice. PSD95 is a scaffold protein that assembles glutamate receptors, ion channel complexes, and signaling proteins. It is significant for regulating synaptic strength and plasticity when receiving patterns of neural signals (Migaud et al., 1998; Carlisle et al., 2008). PSD95 has also been reported to promote the formation of dendritic spines and maintain their stability (Nikonenko et al., 2008; Cane et al., 2014). Therefore, the downregulated PSD95 expression might count for the reduction of dendritic spine density in APP/PS1 mice. SNAP25 is a member of the SNARE family, which plays a significant role in the trafficking of postsynaptic glutamate receptors. In the hippocampus, it takes part in the incorporation of synaptic NMDARs as the target of protein kinase phosphorylation (Lau et al., 2010). In light of the above reports and our results, we speculate that the reduced expression of SNAP25 may also be associated with the NMDAR dysfunction in APP/PS1 mice.

It should be acknowledged that there are some limitations to our research. First, the decreased NMDAR sEPSCs in APP/PS1 mice may not occur in other AD models that present the Tau mutations or mimic the late-onset progression seen in sporadic human AD. Second, our detection of receptor subunit protein expression and electrophysiological research did not distinguish the location of the NMDARs (inside or outside the synapse). Additional studies are necessary to determine the dynamic change of NMDAR function during disease progression and differentiate the changes in the synaptic and extrasynaptic NMDARs in AD models. Altogether, further studies of the destructive effects of NMDAR dysfunction are needed for the development of more effective, mechanism-targeted treatments for AD.

## Data Availability Statement

The original contributions presented in the study are included in the article/supplementary material, further inquiries can be directed to the corresponding authors.

## Ethics Statement

The animal study was reviewed and approved by the Animal Care and Use Committee of the Ningbo University.

## Author Contributions

XZ and LR initiated and designed this study. XZ, LX, HJ, and SY performed the brain slice electrophysiology recordings and analyzed the data. LX and HJ performed the behavioral test. LH and YD performed the Golgi-Cox staining. YZ performed the western blotting. ZL and YJ performed the immunofluorescent staining. LX, YZ, XZ, and HS wrote the manuscript. All authors contributed to the article and approved the submitted version.

## Conflict of Interest

The authors declare that the research was conducted in the absence of any commercial or financial relationships that could be construed as a potential conflict of interest.

## Publisher’s Note

All claims expressed in this article are solely those of the authors and do not necessarily represent those of their affiliated organizations, or those of the publisher, the editors and the reviewers. Any product that may be evaluated in this article, or claim that may be made by its manufacturer, is not guaranteed or endorsed by the publisher.
